# Structure-function analysis of time-resolved immunological phases in metabolic dysfunction-associated fatty liver disease (MASH) comparing the NIF mouse model to human MASH

**DOI:** 10.1038/s41598-024-73150-z

**Published:** 2024-10-03

**Authors:** Anja Schmidt-Christensen, Gustaw Eriksson, William M. Laprade, Behnaz Pirzamanbein, Maria Hörnberg, Kajsa Linde, Julia Nilsson, Mark Skarsfeldt, Diana J. Leeming, Rajmund Mokso, Mariana Verezhak, Anders Dahl, Vedrana Dahl, Kristina Önnerhag, Massoud Rezaee Oghazi, Sofia Mayans, Dan Holmberg

**Affiliations:** 1 Lund University Diabetes Center, Lund University, Lund, Sweden; 2 Department of Physiology and Pharmacology, Karolinska Institutet, Stockholm, Sweden; 3 Technical University of Denmark, DTU, Copenhagen, Denmark; 4 Statistics department, Lund University, Lund, Sweden; 5 Inficure Bio AB, Umeå, Sweden; 6 Nordic Bioscience A/S, Herlev, Denmark; 7 MAXIV laboratory, Lund University, Lund, Sweden; 8 Paul Scherrer Institut, Villigen, Switzerland; 9 Skåne hospital, Malmö, Sweden; 10 Connected Pathology, Lier, Belgium; 11 Department of Medical Biosciences, Umeå University, Umeå, Sweden

**Keywords:** Endocrine system and metabolic diseases, Gastrointestinal diseases, Immunological disorders, Metabolic disorders, Immunology, Diseases

## Abstract

Metabolic dysfunction-associated steatohepatitis (MASH) is a common but frequently unrecognized complication of obesity and type 2 diabetes. The association between these conditions is multifaceted and involves complex interactions between metabolic, inflammatory, and genetic factors. Here we assess the underlying structural and molecular processes focusing on the immunological phase of MASH in the nonobese inflammation and fibrosis (NIF) mouse model and compare it to the human disease as well as other murine models. Histopathology together with synchrotron-radiation-based x-ray micro-computed tomography (SRµCT) was used to investigate structural changes within the hepatic sinusoids network in the NIF mouse in comparison to patients with different severities of MASH. A time-course, bulk RNA-sequencing analysis of liver tissue from NIF mice was performed to identify the dynamics of key processes associated with the pathogenesis. Transcriptomics profiling of the NIF mouse revealed a gradual transition from an initially reactive inflammatory response to a regenerative, pro-fibrotic inflammatory response suggesting new avenues for treatment strategies that focus on immunological targets. Despite the lack of metabolic stress induced liver phenotype, a large similarity between the NIF mouse and the immunological phase of human MASH was detected. The translational value was further supported by the comparative analyses with MASH patients and additional animal models. Finally, the impact of diets known to induce metabolic stress, was explored in the NIF mouse. An obesogenic diet was found to induce key physiological, metabolic, and histologic changes akin to those observed in human MASH.

**Supplementary Information:**

The online version contains supplementary material available at 10.1038/s41598-024-73150-z.

## Introduction

Chronic liver disease encompasses a broad spectrum of etiologies including virus infections, alcohol toxicity, and metabolic stress associated with obesity, type 2 diabetes (T2D) and the metabolic syndrome^1^. The prevalence of metabolic dysfunction-associated steatotic liver disease (MASLD) is increasing steadily, in line with the rising prevalence of obesity^2^. While constituting a significant global health issue and despite the extensive efforts to develop novel and effective treatments, no efficient therapies have yet been identified^3^.

Histologically, MASH is characterized by the presence of steatosis, inflammation, and hepatocyte injury (ballooning)^4^. Most patients with MASLD exhibit only steatosis without further progression^5^, and currently, no reliable biomarkers have been identified that can accurately predict among these patients who will develop MASH, cirrhosis, hepatocellular carcinoma (HCC), and an increased risk of liver-related mortality^6,7^. Although substantial progress has been made in the understanding of the metabolic initiation of MASLD/MASH^8^, the events that trigger the transition into an inflammatory reaction and the development of the immunological phase of MASH are not fully understood. A better understanding of the biological pathways driving the disease is essential for identifying potential therapeutic targets and developing effective treatment. With increasing awareness of the immunological process underlying the progression from benign MASLD to MASH and fibrosis/cirrhosis, potential intervention at this stage of the disease is gaining more attention^9^.

Animal models play a vital role in unraveling MASH pathogenesis and testing therapeutic strategies. Existing models face translation limitations. Our previously developed 2,4αβ.NOD.*Rag2*^−/−^ (NIF) mice spontaneously developing chronic liver inflammation and fibrosis, addresses these issues^10,11^. Here we demonstrate that NIF mice replicate key structural changes seen in human MASH, aligning with immunological processes in later human MASLD/MASH stages. Additionally, we show that obesogenic diets accelerate steatosis, hepatocyte ballooning, and MASH development in NIF mice.

## Results

### The NIF mouse develops liver disease characterized by immunological but not metabolic hallmarks of human MASH

**Fig. 1:**
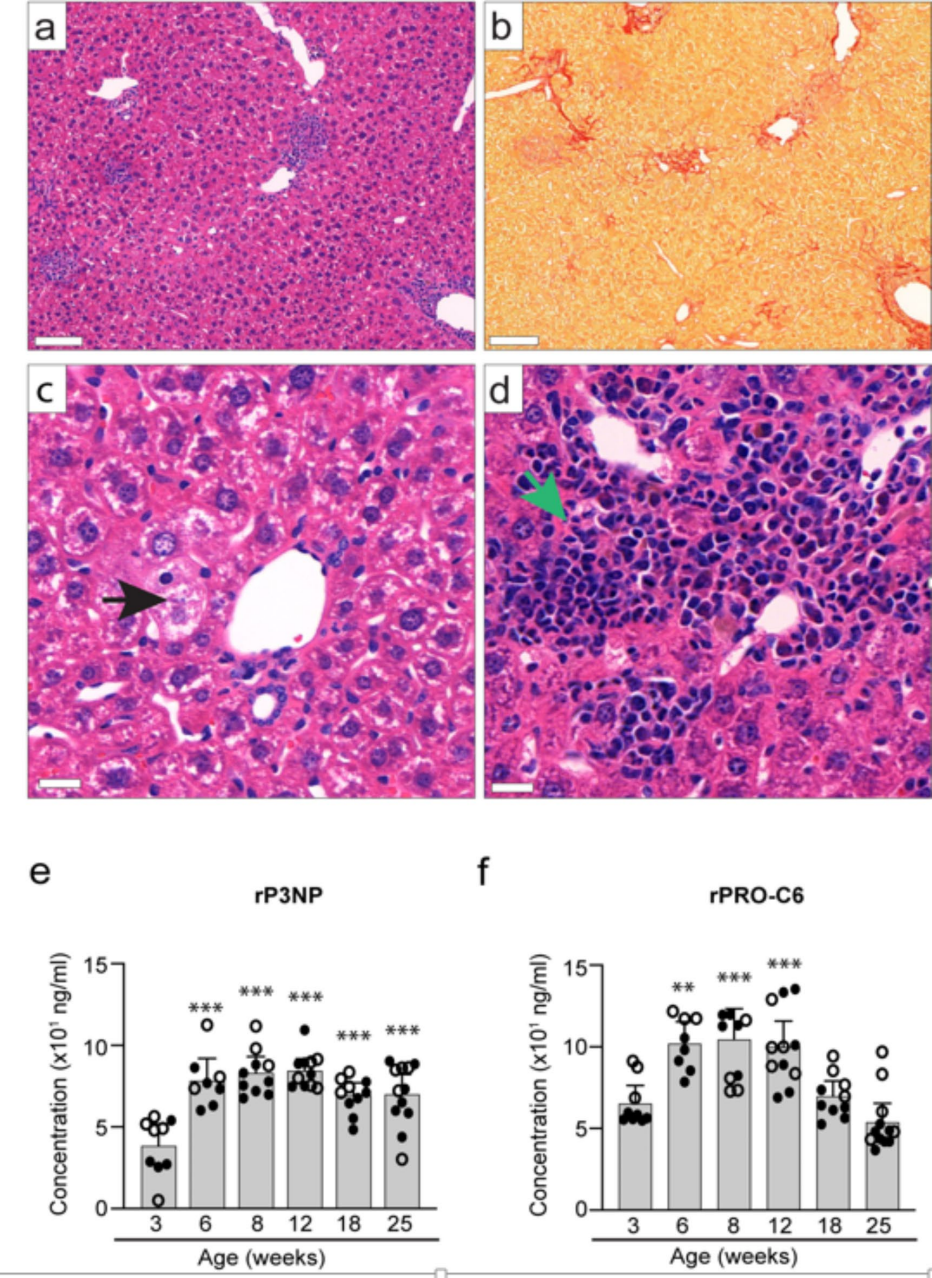
Histopathology in NIF mice. (**a-d**): Liver sections from 8-week-old female NIF mice stained with H&E (**a**,** c-d**) or PSR (**b**). Black arrow (**c**) indicates hepatocyte ballooning and green arrow (**d**) indicates lobular inflammation. Scale bar 100 μm (a, b) or 20 μm (**c**,** d**). (**e**,** f**): Non-fasting serum levels of rP3NP (**e**) and rPRO-C6 biomarkers (**f**) in male (**o**) and female (_•_) NIF mice at different ages (*n* = 8–12 mice/age group). One-way ANOVA was used to compare the mean of the 3-week age group with the mean of the other age groups. Dunnetts post-tests yielded ***p < 0.001 and **p < 0.01, vs. 3-week-old NIF mice.

NIF mice spontaneously develop chronic liver inflammation at approximately 4 weeks, with significant fibrosis evident from 6 weeks, peaking at 8–12 weeks (ISHAK score 3–4), persisting at least until 40 weeks^11^ and Fig. 1a-d). Hepatomegaly is notable from around 6 weeks. Dysregulated glucose metabolism is absent before 18 weeks, but inflammatory cell accumulation and marker upregulation align with hepatitis onset^11^. Despite no dietary triggers, lobular inflammation and consistent hepatocyte ballooning were observed in the majority of animals (Fig. 1c).

Liver fibrosis involves altered extracellular matrix (ECM) dynamics, and biomarkers reflecting ongoing ECM remodeling have proven effective in characterizing fibrotic conditions in humans and animal models^12,13^. To assess their utility in screening anti-fibrotic therapies for NIF mice, we analyzed serum levels of selected markers. Two markers, the rodent pro-peptide of type III collagen (rP3NP) and the endotrophin-associated marker rPRO-C6, correlated significantly with liver fibrosis progression. rP3NP, a known biomarker for tissue repair and fibrosis^14^, showed sustained elevation from 6 to 25 weeks (Fig. 1e). Similarly, rPRO-C6, associated with fibrosis, steatosis, and inflammation, was significantly elevated in NIF mice between 6 and 12 weeks, normalizing thereafter (Fig. 1f). As rPRO-C6 is elevated in advanced fibrosis in MASH patients^15,16^, these findings suggest the potential utility of rP3NP and rPRO-C6 as markers for assessing anti-fibrotic therapies in NIF mice.

### High resolution SRµCT confirms remodulation of microvasculature during progression of liver fibrosis in the NIF mouse

**Fig. 2:**
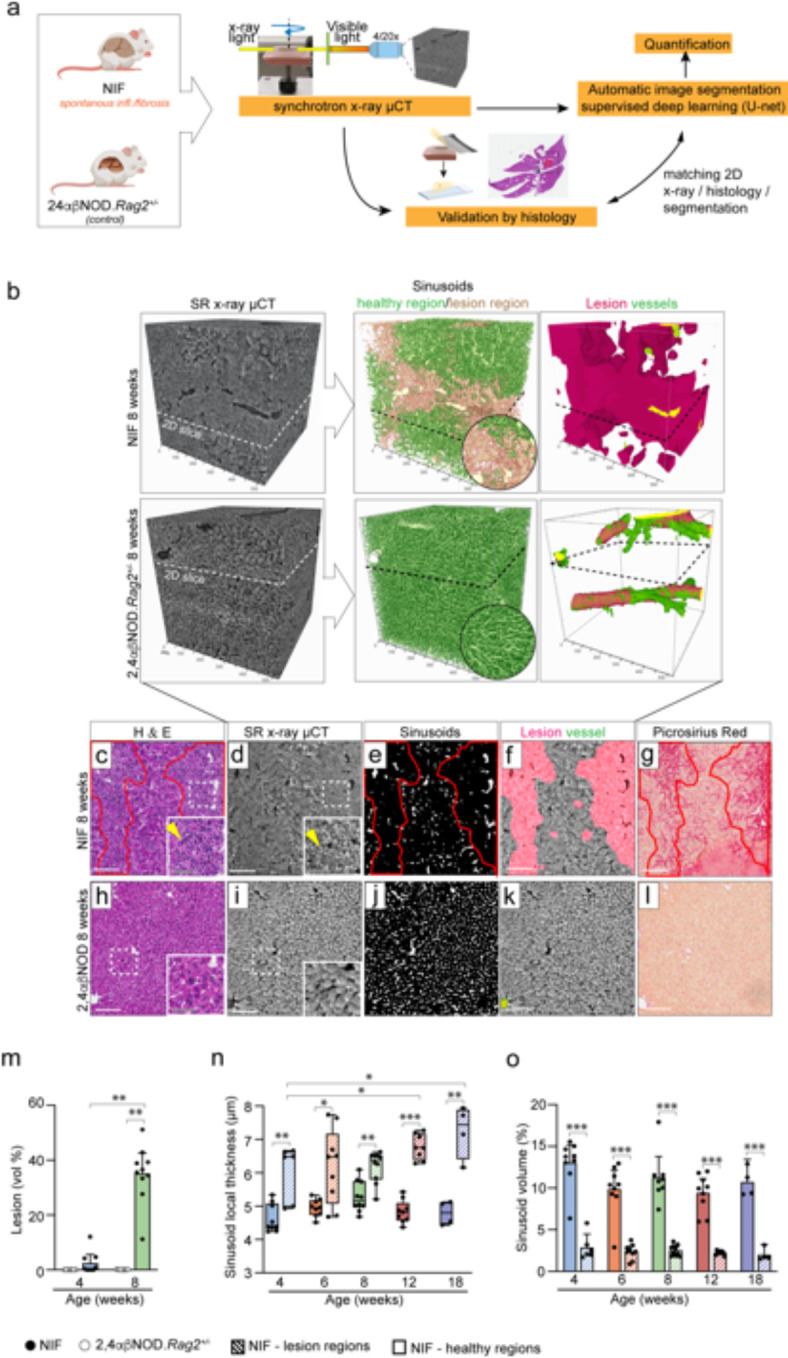
SRµCT reveals microvascular changes in liver lesions of NIF mice. (**a**) Experimental setup: SRµCT imaging of liver samples, validated with 2D histology and guided by deep neural network learning for 3D segmentation. (**b**) Representative SR-µCT scans of 8-week-old NIF and healthy 2,4αβ.NOD.*Rag2*^+/-^ control livers, with 3D volume rendering of segmented features. Sinusoids within lesions are visualized in beige and sinusoids in healthy regions in green. Note: large vessels possess walls with high degree of collagen-fibers, and can be detected by the segmentation tool as minor “lesion” around vessels. (**c-l**): Histology: For validation, scanned liver tissue blocks were sectioned, and consecutive tissue sections were stained with H&E (**c**,** h**) or Picrosirius Red (**g**,** l**) and matched to each acquired dataset with x-ray slices and segmented virtual slices. Dotted lines in the 3D volumes (**b**) refer to the position of virtual 2D sections presented in **d-f**,** i-k**. Red lines in **c** refer to lesion regions guided by histological staining showing disrupted tissue morphology and inflammation (**c**) and fibrosis (**g**). yellow arrow indicates inflammation. Quantification: (**m**) Lesion volume proportions in liver samples of NIF (black circle) and healthy 2,4αβ.NOD.*Rag2*^+/-^ control mice (white circle) at 4 weeks and 8 weeks of age. (**n**,** o**) Sinusoid local thickness (**n**) and sinusoidal volume proportions (**o**) within segmented lesion volumes (fibrotic regions) and adjacent healthy regions in segmented NIF liver volumes at different ages with progressing disease phenotype. Statistical analysis: Lesion volume proportions (**m**) were analyzed using the non-parametric Kruskal-Wallis test with Dunn´s multiple comparisons test for *n* = 4 NIF and *n* = 3 control 2,4αβNOD.*Rag2*^+/-^ with 1–4 scans/mouse. Two-way ANOVA revealed significant effect on phenotype on sinusoid volume proportions (**o**) (*p* < 0,001). Significant interaction between factors was identified for sinusoidal local thickness (**n**) and one-way ANOVA was used instead. Dunnet´s corrections yielded *p ≤ 0.05 comparing the mean of the 4-week age group with the mean of the other age groups of the same phenotype. Additionally, multiple unpaired t-tests with Bonferroni-Dunn multiple comparisons yielded ***p ≤ 0.001, **p ≤ 0.01 or *p ≤ 0.05. comparing healthy regions vs. fibrotic (lesion) regions in NIF livers in different age groups (**n**).

Liver fibrosis in association with MASLD/MASH involves vascular reorganization contributing to clinical complications and liver failure^17^. However, assessing microvascular changes encounters challenges, often relying on limited 3D imaging resolution or 2D histological images, risking loss of crucial encoded 3D information. Synchrotron-based x-ray microtomography (SRµCT) overcomes these limitations, enabling high-resolution 3D imaging of NIF and healthy 24αβNOD.*Rag2*^+/−^ control mouse livers. Employing a deep learning-based segmentation approach, trained using annotated tomographic slices from our custom-build web-based annotation tool and guided by correlative histology we effectively segmented structural features such as lesions, vessels, and sinusoids (Fig. 2a). SRµCT provides micrometer-scale 3D imaging for human biopsies and larger animal tissues (Suppl. Figure 1), offering insights into microvascular changes linked to liver fibrosis. The technique allows virtual dissection (Suppl. Video 1–3) and flexible viewing from any angle, preserving the liver’s volumetric architecture. In 3D visualization, SRµCT revealed fibrotic lesions, vessels, and altered sinusoid structures within lesions of NIF livers distinguishing them from healthy 24αβNOD.*Rag2*^+/−^ control livers (Fig. 2b). Lesions, in this context, refer to regions of tissue with persistent inflammation, fibrosis and disrupted tissue architecture. These features were validated using consecutive histological sections stained with H&E and picrosirius red (PSR) (Fig. 2c, f,g), displaying changes in affected sinusoids, dissolved cellular structures, and disrupted networks in fibrotic regions (Fig. 2b, c-g), clearly distinguishing them from healthy 24αβNOD.*Rag2*^+/−^ controls (Fig. 2b, h-l). Inflammatory cells within lesions were also identified (Fig. 2c, d).

To assess adaptive changes in the sinusoid network following injury and fibrosis, we first measured lesion size (Fig. 2m) and then quantified alterations in the sinusoid network within lesions compared to adjacent unaffected regions within the NIF livers (Fig. 2n, o). Lesion size increased with progressive development of liver fibrosis, starting from a minor proportion in 4-week-old NIF mice, representing early disease onset, and reaching 40% at 8 weeks with full-blown fibrosis (Fig. 2m), consistent with previous characterizations of the NIF mouse model. 24αβNOD.*Rag2*^+/-^ control mice did not develop any lesions, therefore the following analysis concentrated solely on NIF mice. Additionally, we noticed an adaptation in the vascular bed, reflected in increased local sinusoid thickness within lesions over time. Significant differences in sinusoid diameter between affected fibrotic and adjacent unaffected regions became apparent with the onset of lesions at 4 weeks of age and continued to progress over time (Fig. 2n). The proportion of total sinusoid volume within the measured lesions, which varied in size and progressively increased up to 8 weeks of age, was found to be consistently reduced (Fig. 2o).

**Fig. 3:**
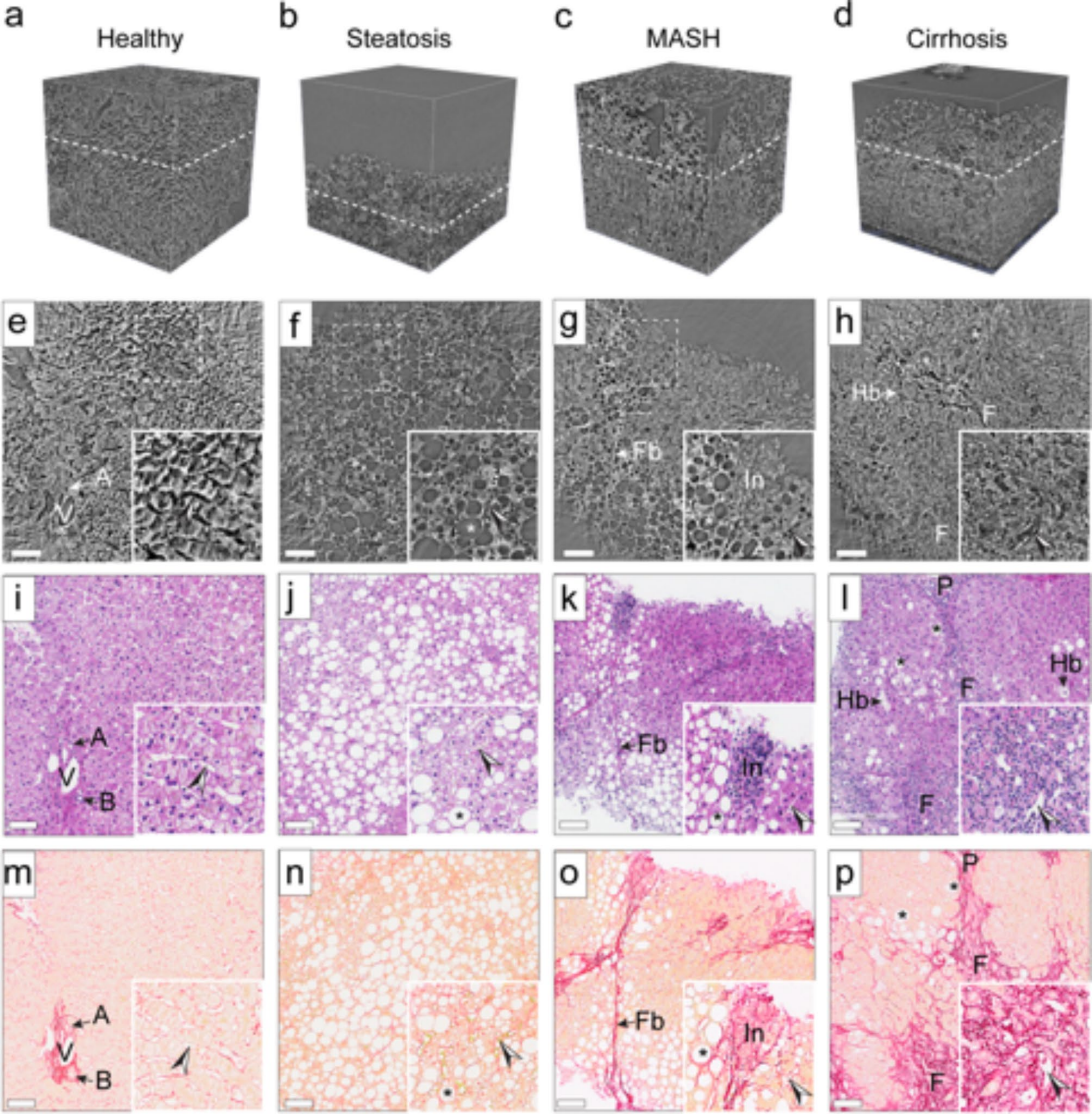
SRµCT imaging of human liver biopsies highlights early MASLD characteristics. (**a-h**) Liver biopsies from healthy volunteers (*n* = 2) and MASLD patients at various stages, - steatosis (*n* = 10), MASH (*n* = 9) or cirrhosis (*n* = 9), underwent high-resolution SRµCT imaging. 3D-renderings (a-d) and 2D cross-sections (e-h), corresponding to histological sections stained with HE (**i-l**) or PSR (**m-p**). Key features labeled: A, artery; B, bile duct; V, vein; F, fibrosis; Fb, fibrous band; In, inflammation; P, portal area; Hb, ballooning hepatocyte; fat droplets are denoted by asterisk, and sinusoids by arrowheads. Scale bar: 100 μm. MASLD assessed for patient samples exemplified in (**a-p**) ranging from NAS = 0 (healthy) to NAS = 7 (cirrhosis), including scoring grades for fat, lobular inflammation, ballooning, and fibrosis: (**i**,** m**) NAS = 0, healthy (0,0,0,0); (**j**,** n**) NAS = 3, steatosis (3,1,0,0); (k, o) NAS = 8, MASH^1–3^ and (l, p) NAS = 7, Cirrhosis^1,4^.

Although the total sinusoid volume within lesions decreased compared to unaffected regions, it remained constant during disease progression SRµCT imaging of human liver biopsies (Fig. 3a-h) unveiled early MASLD features, including macrovesicular steatosis without inflammation and fibrosis (Fig. 3b, f), inflammatory cells and fibrosis in MASH (Fig. 3c, g), and disrupted hepatic architecture replaced by regenerative nodules surrounded by fibrous bands, characteristic of cirrhosis (Fig. 3d, h). These findings align with conventional histology (Fig. 3i-p). Interestingly, liver biopsies from varying degrees of MASLD patients exhibit similar structural changes in the sinusoid network when compared to healthy controls (Fig. 3a, e, i, m).

### Global transcriptome analysis identifying differentially expressed mRNAs associated with the progression of liver disease in NIF mice

**Fig. 4:**
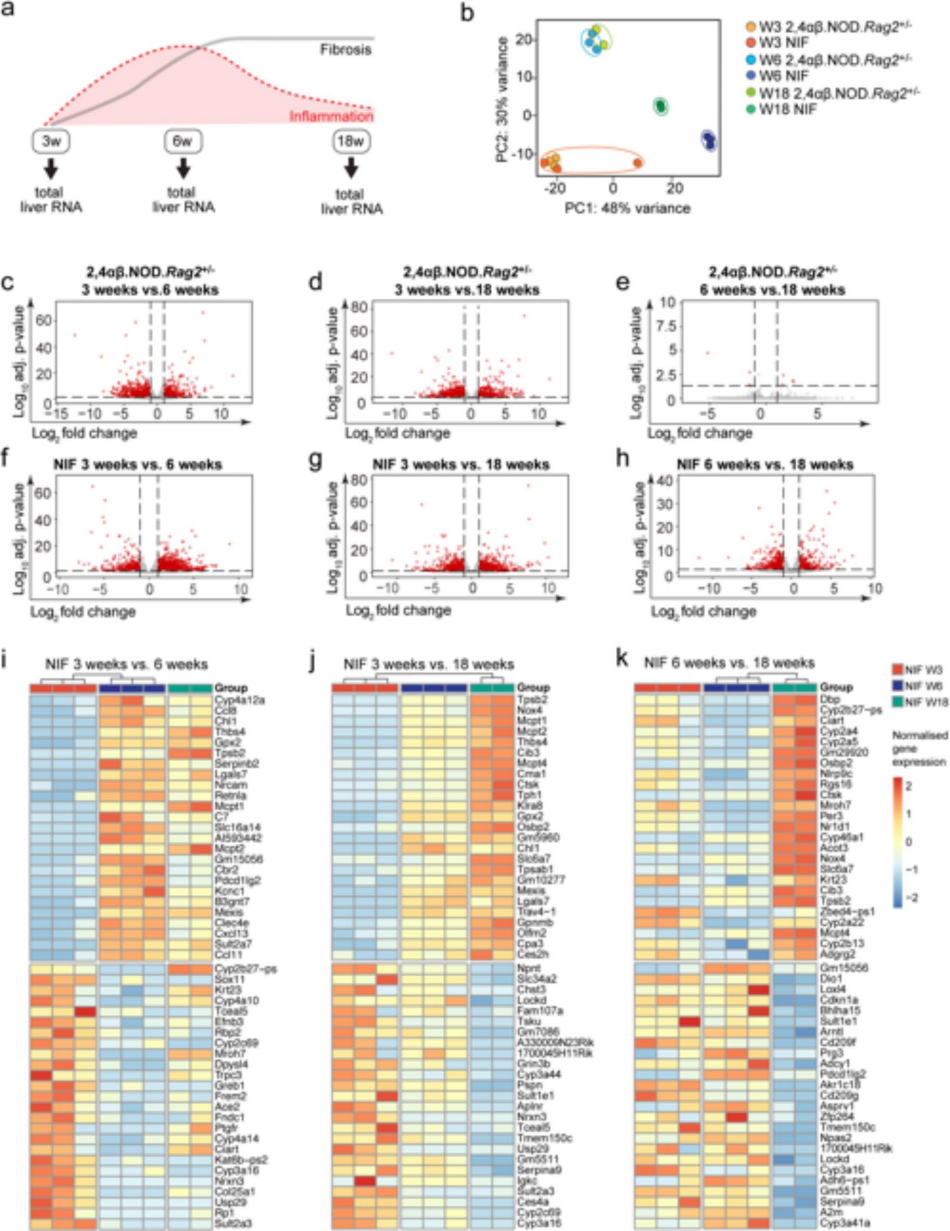
Temporal dynamics of Global liver transcriptomics. (**a**) RNA-seq strategy overview: Libraries prepared from total liver RNA of NIF mice and 2,4αβ.NOD.*Rag2*^+/−^ control mice (*n* = 3/3, *n* = 3/3, *n* = 2/2 mice, for 3 weeks, 6 weeks and 18 weeks, respectively) at indicated timepoints. (**b**) Principal component analysis (PCA), each data point represents an individual mouse. (**c-h**). Volcano plots of differentially expressed genes (DEGs) in 2,4αβ.NOD.*Rag2*^+/−^ control (**c-e**) and NIF mice (**f-h**) comparing 3 vs. 6 weeks (**c**,** f**), 3 vs. 18 weeks (**d**,** g**), and 6 vs. 18 weeks (**e**,** h**). DEGs were identified using DESeq2 default Wald’s test and adjusted by the Benjamini-Hochberg (BH) method. Dashed lines indicate an adjusted p-value < 0.05 and logFC > 1. (**i-k**) Heatmaps show top 25 up- and down-regulated genes in NIF livers at different ages, adjusted p-value cutoff at *p* < 0.05. Each cell represents relative DEG values of normalized variance stabilized transformed gene expression, with samples in columns and genes in rows.

To elucidate molecular processes corresponding to different phases of chronic liver inflammation and fibrosis, we conducted time-resolved next-generation sequencing of hepatic gene expression on poly(A) + mRNA from total liver tissue of NIF and 2,4αβ.NOD.*Rag2*^+/−^ control mice at 3, 6, and 18 weeks (Fig. 4a). Approximately 75.1% of raw sequenced reads uniquely mapped to the mouse reference genome. Differentially expressed genes (DEGs) were identified with BH adjusted p-value < 0.05 and log2FC > 1 (Suppl. Figure 2, Suppl. Tables 1–5, Suppl. Figure 5). Principal component analysis (PCA) (Fig. 4b) and Euclidean distance analysis (Suppl. Figure 2) revealed distinct separation between NIF and 2,4αβ.NOD.*Rag2*^+/−^ control mice at 6 and 18 weeks. However, 3-week-old NIF and 2,4αβ.NOD.*Rag2*^+/−^ control mice clustered together but distinctly from 6- and 18-week-old counterparts (Fig. 4b). Notably, 6- and 18-week-old NIF mice formed separate clusters, while 2,4αβ.NOD.*Rag2*^+/−^ mice of these age groups clustered together. Volcano plots (Fig. 4c-h) and Venn diagrams (Suppl. Figure 3) illustrated differentially expressed mRNAs. Comparing 2,4αβ.NOD.*Rag2*^+/−^ animals of different age groups revealed 1173 DEGs between 3- and 6-week-olds (Fig. 4c, Suppl. Figure 3a) and 1356 DEGs between 3- and 18-week-olds (Fig. 4d), with 900 DEGs common in both comparisons, indicating expected developmental changes. Conversely, only 8 DEGs were identified when comparing 6- and 18-week-old controls (Fig. 4e Suppl. Figure 3a).

Comparing NIF mice within the same age groups (Fig. 4f-h), we filtered out DEGs identified in 2,4αβ.NOD.*Rag2*^+/−^ control group comparisons to account for age-related changes. Following this correction, 1614 DEGs were identified in 3- vs. 6-week-old (Fig. 4f) and 1398 DEGs in 3- vs. 18-week-old NIF mice (Fig. 4g). Notably, in contrast to 2,4αβ.NOD.*Rag2*^+/−^ groups, the comparison of 6- and 18-week-old NIF mice revealed a substantial 1245 DEGs (Fig. 4h and Suppl. Figure 3b). A heat map of top up- and down-regulated genes in NIF mice at these time points highlighted a temporal shift in gene expression corresponding to observed histopathological changes in liver pathology (Fig. 4i-k).

### Temporal liver transcriptomics analysis aligns time-resolved patterns of type 1 and type 2 inflammatory responses

**Fig. 5:**
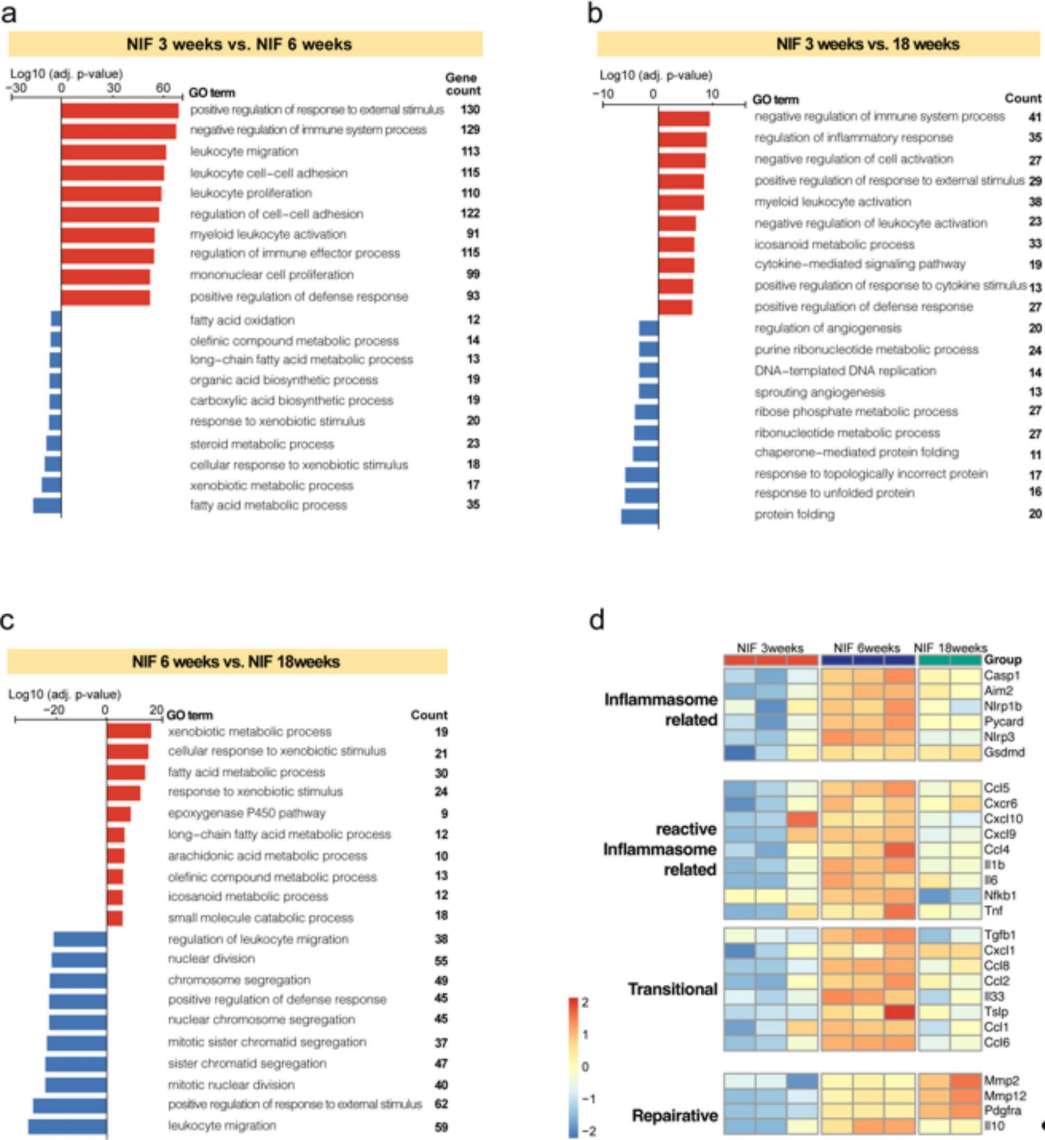
Temporal gene expression shift in NIF mouse model. **(a-c**) GOEA assessing gene expression in liver tissues of NIF mice at 3, 6, and 18 weeks (*n* = 3 mice, *n* = 3 mice, *n* = 2 mice, respectively). Top 10 enriched pathways and gene counts for 3 vs. 6 weeks (**a**), 3 vs. 18 weeks (**b**), and 6 vs. 18 weeks (**c**) are displayed. Rank gene sets are based on significance (Log10, *p* < 0.05) after age-effect corrections. Orange and green indicate upregulated and downregulated gene expression. (**d**) Heat map of selected DEGs illustrating temporal regulation related to the inflammasome and indicated inflammation phases. Each colored cell shows relative DEG values defined by normalized variance stabilized transformed gene expression and compared to other ages.

We next conducted Gene Ontology Enrichment Analysis (GOEA) on DEGs identified across different age groups of NIF mice. Results were filtered based on an adjusted p-value < 0.05 and > 3 enriched genes in pathways. The NIF mouse lacks the expression of the Rag2 gene. To minimize the effect of the presence/absence of Ig and TCR genes on the analysis, DEGs were filtered to exclude these genes from the further analysis. Figure 5 presents the top 10 significantly upregulated and downregulated pathways from GOEA when comparing 3- and 6-week-old animals and changes between 6- and 18-week-old animals. This highlights dynamic hepatic gene expression changes associated with liver disease progression in the NIF mouse model. Pathways upregulated between 3 and 6 weeks (Fig. 5a, Suppl. Table 3a) and between 3 and 18 weeks (Fig. 5b, Suppl. Table 3b). were predominantly related to immune response and inflammation, while downregulated pathways were linked to metabolism. Conversely, comparing 6 to 18 weeks in NIF mice (Fig. 5c, Suppl. Table 3c), top upregulated pathways were mainly related to metabolism, and prominent downregulated pathways included those involved in leukocyte migration and cell proliferation.

The observed liver transcriptional program dichotomy in NIF mice at 6 and 18 weeks aligns with a progressive liver disease peaking in reactive inflammation at 6 weeks and transitioning to a reparative/profibrotic phase at 18 weeks. Previous studies on NIF mouse liver inflammation indicated an initial inflammasome pathway activation leading to a reactive inflammatory response^11^. Consistent with this, genes associated with the inflammasome were upregulated at 6 weeks but downregulated at 18 weeks (Fig. 5d). Similar expression patterns were noted for genes linked to reactive inflammatory responses and the transition to a reparative phase. Some inflammation-related genes remained upregulated at 18 weeks (*Cxcl1*,* Ccl22*,* Pdgfra*,* Mmp2*,* Mmp12*), consistent with a type 2 reparative, pro-fibrotic response. This data suggests that liver fibrosis development in NIF mice involves an initial reactive inflammatory phase transitioning into a regenerative, pro-fibrotic, inflammatory response. In addition to GOEA, Gene Set Enrichment Analysis (GSEA) was performed on the same DEG’s as for GOEA (Suppl Fig S3a-c, Suppl table 4a-c), revealing similar patterns as with GOEA.

### Comparative transcriptomics reveals similarity to human MASH

**Fig. 6:**
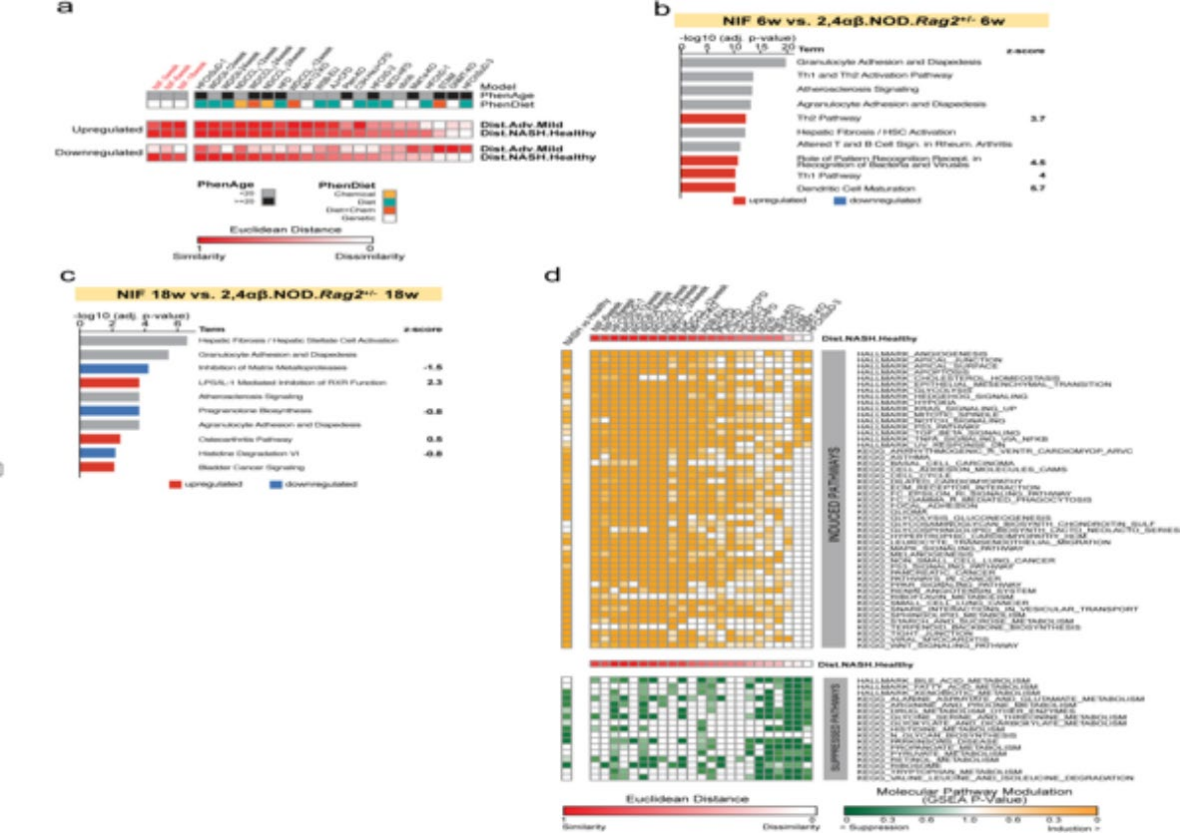
Comparative hepatic gene expression in NIF mice, human MASH- and other mouse models for MASH. (**a**) Heat map comparing human MASH liver transcriptomes (advanced vs. mild MAFLD, MASH vs. healthy) with NIF mice at 3, 6, and 18 weeks, and 16 other MASH mouse models (specified in Suppl. Table 5). Similarity assessed using GSEA and Euclidean distance, with red indicating FDR values for transcriptome similarity. (**b**,** c**) Top IPA canonical pathways in NIF liver at 6 (b) and 18 weeks (**c**) with z-scores predicting pathway activation (positive) or inhibition (negative). Grey bars denote pathways with zero or close to zero z-score or ineligibility for prediction. Ranked gene sets based on significance (Log10, *p* < 0.05), excluding immunoglobulin and T cell receptor genes. (**c**) Comparative hepatic gene expression in NIF mice, human MASH-associated gene and other mouse models for MASH. Heat map of cross-species sample Similarity of human MASH liver transcriptome datasets (MASH patients vs. healthy individuals) vs. NIF 6 weeks, NIF 18 weeks and a panel of 16 previously published diet, chemical, and/or genetic MASH mouse models^26^ with statistically significant dysregulation defined as FDR < 0.01 in either of the two human MASH liver transcriptome datasets: advanced vs. mild MASLD patients (GSE49541), and MASH patients vs. healthy individuals (GSE48452). Dysregulation of the selected gene sets was similarly determined by GSEA in a panel of 16 previously published diet, chemical, and/or genetic MASH mouse models (Suppl. Table 1), Dysregulation of the selected gene sets was determined by GSEA and similarity to the human datasets was determined by Euclidean distance. Red color on heat map indicates FDR values for the transcriptome similarity. Top ranked induced (orange) or suppressed (green).

To assess the translational relevance of the NIF mouse model, we compared the dataset obtained from the NIF mouse with publicly available datasets from other mouse models (Suppl. Table 5) for liver disease and two human datasets representing comparisons between MASH patients versus healthy controls and advanced versus mild MASH^18^. Applying the same GSEA approach as Friedman et al.^18^ for direct comparison, we calculated the transcriptomic similarity of the NIF mouse dataset to human MASH and various mouse models. This analysis revealed a high degree of similarity in gene expression patterns between human MASH and the NIF mouse (Fig. 6a), identifying pathways closely corresponding to the data displayed in Fig. 5 (Fig. 6b, c, d).

### An obesogenic diet and metabolic stress induce steatosis and hepatocyte ballooning in NIF mice

**Fig. 7:**
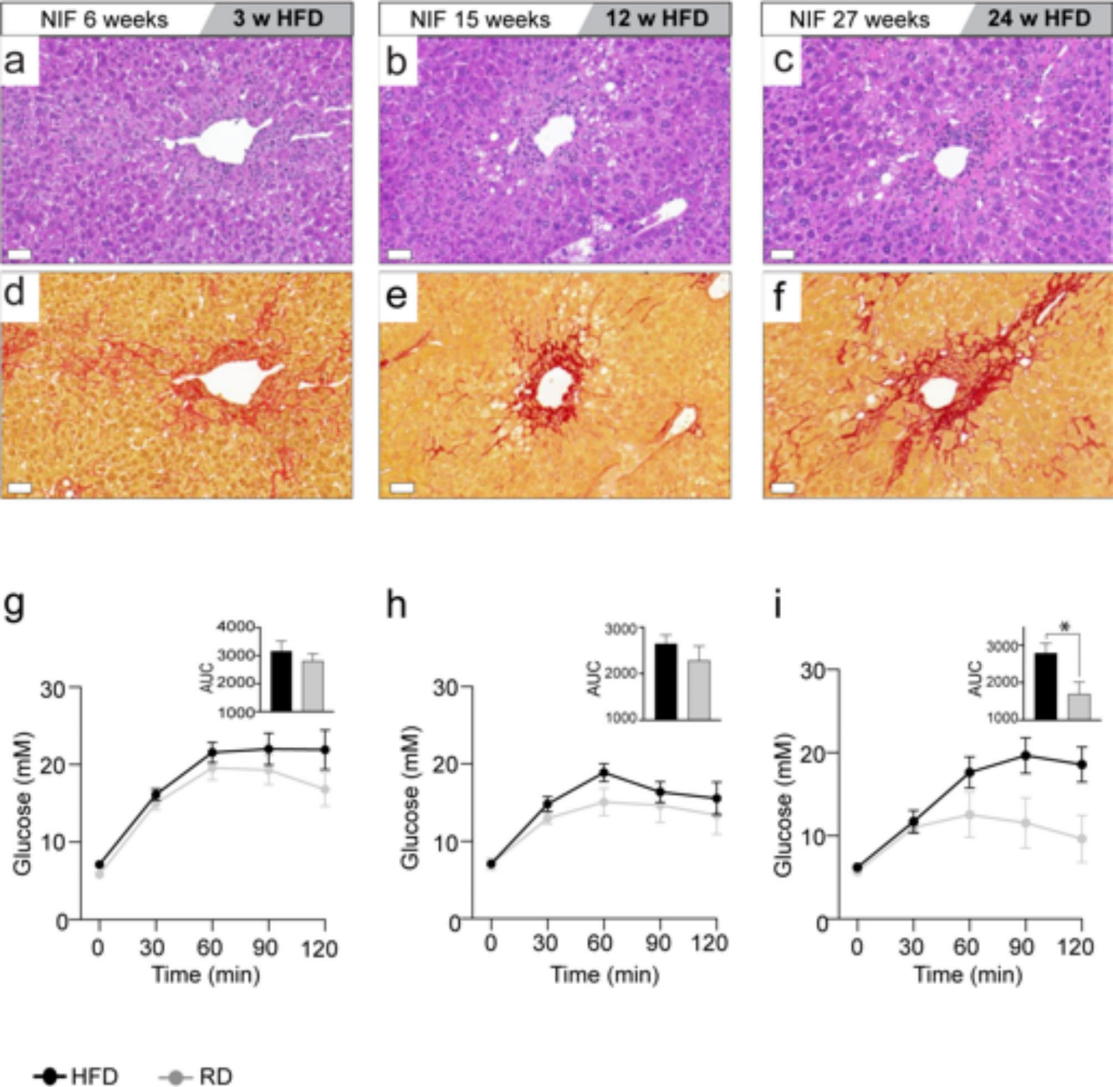
HFD impact on NIF liver pathology and glucose metabolism. (**a-i**) Six-week-old male NIF mice were fed HFD or normal diet for 3, 12, and 24 weeks (*n* = 10/10, *n* = 10/8, *n* = 6/7 mice, respectively). Liver sections stained with H&E (**a-c**) and PSR (d-f) at each endpoint. OGTT assessed glucose metabolism at 3 weeks (**g**), 12 weeks (**h**) and 24 weeks HFD (**i**). Mean with SEM is shown and inserts display area under the curve (AUC) from OGTT. Significance determined by unpaired two-tailed t-test (*p < 0.05 vs. normal diet, 95% CI).

Despite the absence of induced metabolic stress in the NIF mouse, the observed similarity in liver gene expression patterns with human MASH suggested a shared inflammatory process. To explore whether metabolically provoking the NIF mice would impact inflammation, we subjected them to a high-fat diet (HFD, 60% energy from fat) for 6, 12, or 24 weeks (Fig. 7a-f). Altered glucose metabolism in NIF mice became apparent after 6 weeks on HFD (Fig. 7g), intensifying after 12 (Fig. 7h) and 24 weeks (Fig. 7i). Ultimately, HFD-fed NIF mice exhibited fatty liver and steatohepatitis, serving as a murine model mirroring several features of human MASH.

**Fig. 8:**
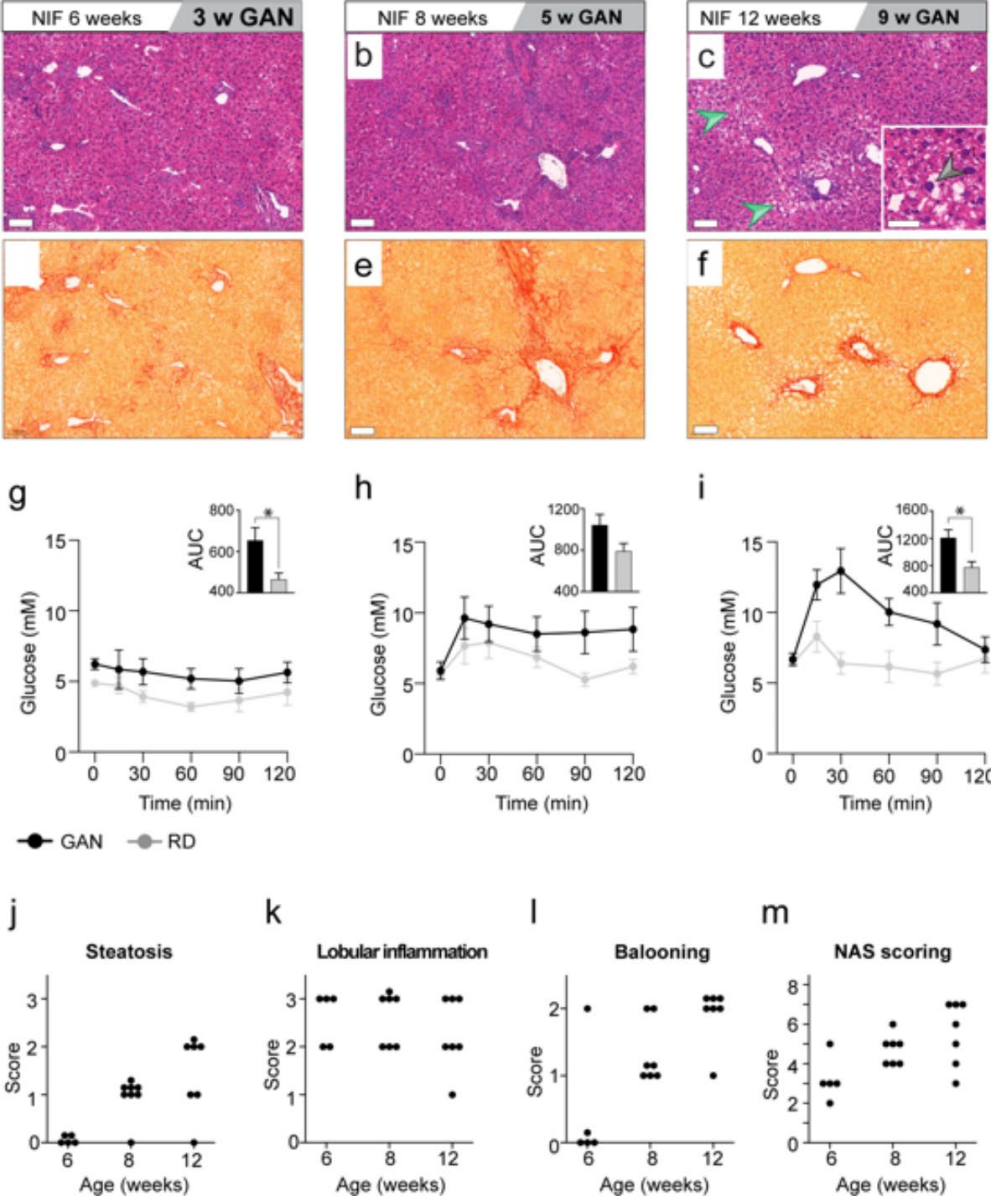
GAN diet impact on NIF liver pathology and glucose metabolism. (**a-m**) Male NIF mice were fed GAN diet or normal chow for 3, 5 and 9 weeks (*n* = 5/5, *n* = 7/7, *n* = 7/7 mice, respectively). Liver sections were stained with H&E (**a-c**) or PSR (**d-f**). Green arrow indicates steatosis, and black arrows shows hepatocellular ballooning degeneration. Scale bars: 100 μm (**a-f**) or 50 μm (insert in **c**). OGTT assessed glucose metabolism at 3 weeks (**g**), 5 weeks (**h**) and 9 weeks GAN diet (**i**). Data shown as mean with SEM, and significance was determined by unpaired two-tailored t-test, *p < 0.05 vs. normal diet, 95% CI). GAN-fed animals were scored for steatosis (**j**), Lobular inflammation (**k**) and hepatocyte ballooning (**l**) according to the NAFLD Activity Score (NAS) system. The summed NAS score is indicated in (**m**).

The Gubra Amylin NASH (GAN) diet induces a MASH phenotype in C57Bl/6J and ob/ob mice^19^. To assess its impact on NIF mice, we administered the GAN diet for 3, 6, or 9 weeks (Fig. 8). H&E and picrosirius red (PSR) staining of collagen fibers revealed accelerated MASH-related histopathological changes compared to HFD (Fig. 8a-f). SRµCT imaging provided complementary insights into 3D structural alterations within the liver (Suppl. Figure 4). GAN-fed mice showed impaired OGTT results from the third week (Fig. 8g-i). All GAN-fed mice developed steatosis, inflammation, and hepatocyte ballooning, progressing to borderline or full-fledged MASH (≥ 5 NAS) by 8 weeks (Fig. 8a-f, j-m). NAS increased after 3 weeks on GAN diet (Fig. 8j-m), with inflammation playing a prominent role in earlier stages and later stages involving steatosis, inflammation, and ballooning, resulting in consistently higher NAS compared to chow-fed mice.

## Discussion

Identifying individuals with MAFLD at risk of developing severe liver complications poses a significant challenge but holds potential medical and societal rewards. A key component in the pathophysiology of these severe complications is the role of the innate immune system^1^. Enhancing our understanding of the cellular and molecular interactions within the innate immune system will improve our comprehension of the pathogenesis of the disease and provide tools for identifying patients at risk of developing complications. Animal models that accurately reflect these specific aspects of the human disease are rare but constitute valuable tools in these efforts. In this study, we employed the NIF mouse model, mirroring sterile liver inflammation and fibrosis, to highlight its substantial similarities to the immunological phase of human MASH at both the histopathological and tissue structure levels, demonstrated through high-resolution synchrotron-based X-ray tomography.

The time-resolved transcriptomics analysis demonstrated that the gene expression patterns varied together with the liver disease phenotype in the NIF mouse and implied a progression from a largely unaffected liver at 3 weeks through an early inflammatory phase characterized by a reactive, predominantly type 1 inflammatory response evident at 6 weeks of age. Subsequently, a late phase emerges, characterized by a regenerative, pro-fibrotic, type 2 inflammatory response. These findings lead us to hypothesize that the progression of the immunological phase in human MASH may follow a similar trajectory. Supporting this hypothesis, both reactive and regenerative inflammatory responses have been reported in human MASH^20,21^ and the balance between type 1 and type 2 inflammatory responses is suggested to be critical in the development and progression of liver fibrosis^22^.

Research has indicated that type 1 cytokines are elevated in patients with liver fibrosis and the administration of Th1 cytokines has been shown to exacerbate the fibrosis progression. In contrast, blocking the activity of these cytokines can diminish fibrosis and improve liver function. On the other hand, type 2 cytokines, particularly IL-13, have been recognized for their role in activating fibroblasts and promoting extracellular matrix deposition^23^ which are key components in development of human MASH^24^. Thus, a skewing towards either type 1 or type 2 responses can promote fibrosis, pointing to the potential necessity of therapies that concurrently targets both types of responses in order to effectively treat liver fibrosis^20^. The NIF mouse model provide a distinctive tool to investigate these therapeutic approaches.

This study utilizes high-resolution imaging to analyze the liver microstructure changes during fibrosis progression. Increasing scarring and extracellular matrix deposition, indicative of advancing fibrosis and measured lesion size, correlate with increased local sinusoid thickness and consistent reduced total sinusoid volume, specifically confined to lesions. Our findings support existing literature^25^ suggesting fibrosis disrupts liver architecture, causing structural and functional modifications in the sinusoids. Unlike previous studies focusing on molecular and cellular aspects of liver microcirculatory dysfunction, our research provides new 3D insights into sinusoids and fibrotic lesion microstructures. Our novel image analysis approach´s strength lies in the application of deep learning to 3D SRµCT images with cellular resolution, particular for liver fibrosis. This allows us to measure lesion size and differentiate vascular structural changes in segmented sub-volumes (lesion vs. non-lesion regions) as early as initial lesions appear at 4 weeks of age. This allows for quantification of volumetric parameters early in the disease, serving as potential fibrosis-related biomarker, even in small sample sizes like needle biopsies, providing valuable insights into human MASH. It´s important to note that in this context, the Artificial intelligence (AI) segmentation method is trained by experts and guided by correlative histology. It identifies learned patterns of established lesions rather than evolving disease patterns. Therefore, we cannot rule out a gradual reduction of sinusoidal volume within the early disease stages that might go undetected by our method. Analyzing fixed tissue limits our ability to directly assess implications for blood flow and pressure. However, as smaller vessel volumes in fibrotic lesions accommodate the same blood volume and lesion size increases with progressing disease, we hypothesize a potential contribution to sinusoidal hypertension, leading to progressive liver damage. Our study suggests that alterations in the sinusoid network during fibrosis may impact disease progression, offering potential therapeutic avenues. Quantifying functional properties such as permeability and fenestration would further enhance our understanding of sinusoidal dysfunction in the NIF model. Future studies incorporating live imaging techniques and functional assays will be essential to comprehensively assess these features and their implications for liver microcirculatory dysfunction. In summary, our work underscores the significance of high-resolution imaging techniques for understanding complex tissue microstructure and its dynamic changes in disease.

Our comparative transcriptomics analyses have confirmed, at the level of gene expression, the similarity of the NIF mouse model to the immunological phase of human MASH. We have previously reported on how this model can be efficiently used to separate therapeutic effects that target the inflammatory phase of MASH from effects mediated by targets involved in the metabolic phase of the disease. In this study, we demonstrate that the hepatic gene expression profile in the NIF mouse closely mirrors that of human MASH patients. The fact that NIF mice develop liver disease spontaneously, without the need for diet-induced metabolic stress, suggests that the observed similarities are predominantly reflective the immunological aspects of MASLD/MASH progression. This is further substantiated by pathway and enrichment analyses of the liver of NIF mice at 6 and 18 weeks of age, which revealed a time-resolved regulation of inflammation aligned with the analogous process in MASH.

While dietary models for MASH have often been favored over genetic models, these can be time-consuming and may not always results in severe steatohepatitis and advanced fibrosis^26^. Toxic models, particularly the Carbon tetrachloride (CCl4)-induced liver fibrosis have been popular, yet they tend to mimic acute rather than chronic liver inflammation and fibrosis, which is more representative of human disease^27^. More recent approaches, such as a Western diet (WD), high-fat, high-fructose and high-cholesterol combined with CCl4 treatment have shown considerable parallels to human NAH in both histopathological and comparative transcriptomics profiling^28^. Our findings demonstrate that the NIF mouse model can be extended to encompass the metabolic phase of the disease when administered obesogenic diets. In this model, both the classic HFD and the more recent GAN diet induced steatosis, hepatocyte ballooning and MASH in the NIF mouse. GAN diet led to a more accelerated progression of liver disease that manifested major hallmarks of MASLD and MASH, thus capturing the entire spectrum of both metabolic and inflammatory phases of the disease development.

## Methods

### Human tissue samples

Human liver tissue samples were obtained from Regional Biobank Centrum Syd. Ethical approval (Regional Biobank Centrum Syd, Dnr 2019–06302) was obtained to collect paraffin-embedded liver biopsies from patients with steatosis (*n* = 10), MASH (*n* = 9), cirrhosis (*n* = 9), or controls (*n* = 2) through the Department of Clinical Pathology, Skåne University Hospital Malmö/Lund.

### Animal experiments

The study was reported in accordance with the ARRIVE guidelines and animal experiments strictly adhered to Swedish Board of Agriculture guidelines, approved by the Umeå University ethics committee (permit no A3-2018). The mice were euthanized by cervical dislocation. All efforts were made to minimize animal suffering. Animals were housed in specific pathogen–free conditions at Umeå University’s animal facility in Sweden. Experimental animals were randomly selected from the breeding colony in regards to litter and cage. However, genotyping was necessary to assign the mice to their respective experimental groups. The nonobese diabetic inflammation and fibrosis (NIF) mouse model for liver fibrosis has been previously reported^10,11^. To generate 2,4αβNOD.Rag2−/− (NIF) mice, 2,4αβNOD.Rag2−/− male mice were bred with 2,4αβNOD.Rag2+/− female mice, producing both NIF (2,4αβNOD.Rag2−/−) and control (2,4αβNOD.Rag2+/−) offspring. The immunodeficient NIF mice (2,4αβNOD.Rag2−/−) develop chronic inflammation and fibrosis in several organs, including the liver, while the control mice (2,4αβNOD.Rag2+/−) do not. The NIF mouse model exhibits spontaneous inflammation and fibrosis, beginning at 3–4 weeks of age and escalating in severity until 6–8 weeks of age. The severity of these lesions increased in the 8- and 12-week-old NIF mice, after which the progression halted. Both male and female mice have been used to characterize key processes associated with the pathogenesis in the NIF mouse (Figs. 1, 2, 3, 4, 5 and 6), while only male mice have been used for dietary intervention experiments (Figs. 7 and 8).

### Dietary interventions

Following weaning, male NIF mice from several litters were maintained on either a high-fat diet (HFD; 21.9 kJ/g [5.24 kcal/g], 34.9% (wt/wt) fat, 26.2% (wt/wt) protein, 26.2% (wt/wt) carbohydrate; D12492, New Brunswick, NJ, USA), Gubra Amylin NASH diet, (GAN diet; 40 kcal% Fat (Mostly Palm Oil), 20 kcal% Fructose and 2% Cholesterol D09100310, Research Diets, New Brunswick, NJ, USA), or a normal diet (ND; 12.6 kJ/g [3 kcal/g], 4% (wt/wt) fat, 18.5% (wt/wt) protein, 55.7% (wt/wt) carbohydrate; R36, Lactamin AB, Stockholm, Sweden). From each litter, mice were selected in a consequtive order to receive either of the diets until ech time point/diet group reached *n* > 5.

### Histopathological analysis

Mice were euthanized by cervical dislocation, and livers were perfused with PBS via the inferior vena cava. Liver tissue were fixed in 4% neutral buffered formalin, embedded in paraffin, sliced into 3 μm sections, and stained with hematoxylin & eosin (H&E) to evaluate liver morphology or picrosirius red (PSR) (Sigma, 365548-5G) to assess fibrosis. An ECVP board certified pathologist used a light Leitz Diaplan microscope to assess H&E and PSR-stained liver sections according to a classical 5-point semiquantitative scoring scale of the lesions. In addition, a six-point Ishak scoring scale was used to evaluate the PSR-stained liver sections. The NAFLD Activity Score (NAS) was determined by an expert pathologist using the NASH CRN scoring system^29^. All histological scoring was performed in a blinded manner.

### Synchrotron radiation-based microtomography (SRµCT)

Formalin-fixed paraffin-embedded liver tissue blocks underwent synchrotron-based microtomography (SRµCT) at the TOMCAT beamline, Swiss Light Source (SLS). Two to three randomly selected regions were scanned for each of the 36 liver specimens, including 2–4 NIF (*n* = 4 for 4 weeks, 6 weeks, 8 weeks and 12 weeks, *n* = 2 for 18 weeks of age) and 3 control female or male 2,4abNOD.*Rag2*^*+/−*^ mice per age group. Using monochromatic photons (21 keV), transmitted X-ray photons were converted into visible light via a 20-µm LuAG: Ce scintillator screen, coupled to a sCMOS detector (2160 × 2560 pixels) with 1.6 μm (4x) and 325 nm (20x) effective pixel sizes. Paganin et al.´s algorithm^30^ was employed for phase retrieval.

### Processing of synchrotron radiation-based microtomography (SRµCT) data

Volumes at 20x magnification were cropped to 1600 × 1600 × 1600 and downscaled to 800 × 800 × 800 via 2x binning for computational efficiency without accuracy loss. Linear model correction addressed bias fields, masked intensity reduction minimized bright artifacts, and volume intensities were normalized. Preprocessed volumes were input into a custom-build browser-based annotation tool that utilizes deep learning to facilitate efficient data labeling and image segmentation, differentiating between sinusoid and non-sinusoidal, vessel and non-vessel, and lesion and unaffected regions The Training of the model was done by experts using histology as cross-reference as described previously^31^. Quantification: The proportion of lesion in NIF and 2,4abNOD.*Rag2*^*+/−*^ control mice is determined by dividing the number of lesion voxels by the total volume, excluding vessels. To quantify the proportion of sinusoidal volume in NIF mice within lesion regions, the volume of sinusoids within the lesion regions is compared to the total volume of the lesions themselves. A similar approach is applied in unaffected regions. The local thickness is computed as, for each voxel within a 3D object, it is assigned the radius of the largest sphere that can fit entirely within the object while encompassing the voxel. Visualization was performed using two software packages: Imaris (Bitplane AG, version 9.1, Zurich) and Tomviz (Kitware, Inc., version 1.10.0, Clifton Park, NY, USA).

### Serum markers

Serum was obtained by centrifugation (10 min at 1500 x g) from clotted whole blood. Collagen turnover markers were assessed at Nordic Bioscience: rodent P3NP (rP3NP) for collagen type III formation using a CLIA assay technology and specific antibody (Nordic Bioscience Catalog # NBH161 lot 2203 A). rP3NP was quantified in 4-fold diluted serum. Collagen type VI levels were measured via an ELISA assay using rodent PRO-C6 (rPRO-C6) (Nordic Bioscience, Catalog #1033, lot 1033 A)^16^ in undiluted serum samples. All analysis of serum markers was performed in a blinded manner.

### Cytokine protein analysis

Plasma was collected using EDTA-coated microcentrifuge tubes and centrifuged for 15 min at 1000 x g. The cleared plasma was collected and stored at − 80 °C before being analyzed for cytokines using the MSD U-PLEX platform (Meso Scale Diagnostics, Rockville, MD, USA) according to the manufacturer’s instructions.

### Glucose tolerance tests

Oral glucose tolerance tests (OGTTs) were performed in overnight-fasted mice at the end of the study. Mice were administered a glucose solution (2 g/kg body weight) by oral gavage, and blood glucose levels were measured at 0, 15, 30-, 60-, 90- and 120-minutes post-gavage using a glucometer (AlphaTrak 2, Zoetis). Area under the curve (AUC) for glucose was calculated from the glucose values obtained during the 120-minute OGTT.

### RNA-sequencing and data processing

Global transcriptome profiling of the mouse liver was performed by RNA-Seq. Briefly, a sequencing library was prepared using total RNA by TruSeq library prep kit (Illumina) following manufacturer’s protocol, and resulting data were preprocessed by Genevia OY custom preprocessing pipeline. TrimGalore (version 0.6.4)^32^ was used for quality and adapter trimming in paired-end mode using Cutadapt (version 2.4)^33^ with PHRED score cut-off set to 20 and reads > 20 base pairs in length with an error rate < 10%. Quality control on raw and trimmed reads was performed with FastQC (version 0.11.8)^34^ Trimmed reads were aligned to the mouse reference GRCm38.96 using STAR aligner (version 2.7.3)^35^ with the quant mode generating the gene count matrix for downstream analysis. The datasets generated and analyzed during the current study are available in the Gene Expression Omnibus (GEO) (GSE25526).

### Statistical and bioinformatics data analysis

Results are presented as the means ± 95% confidence interval (CI) unless specified otherwise. Statistical analysis was conducted using GraphPad Prism v.10 software (San Diego, CA). The Shapiro-Wilk test was used to assess the normality of data distribution and to identify potential outliers. Significance levels were determined as specified in the text using one- or two-way ANOVA or multiple unpaired two-tailored student’s t-test, followed by Dunnett’s or Bonferroni multiple comparison tests. The non-parametric Kruskal-Wallis test followed by Dunn’s multiple comparison test was used when data was not normally distributed or sample sizes were too small. In total, 60 mice were studied for the biomarker analysis (Fig. 1) with a minimum of 8 mice in each group, 36 mice for the SRµCT imaging study (Fig. 2) with a minimum of 3 mice in each group (exception for 2 NIF mice in 18 weeks age group), 16 mice for the gene expression analysis (Figs. 4, 5 and 6) with a minimum of 3 mice per group (3 and 6 weeks) or 2 mice per group (18 weeks) and 89 mice for the dietary interventions (Figs. 7 and 8) with a minimum of 5 mice per group.

Differential gene expression utilized DESeq2^36^ with four contrasts used for comparing differential expression (2,4αβ.NOD.*Rag2*^+/−^ week 3 vs. 2,4αβ.NOD.*Rag2*^+/−^ week 6, 2,4αβ.NOD.*Rag2*^+/−^ week 6 vs. 2,4αβ.NOD.*Rag2*^+/−^ week 18, NIF week 3 vs. NIF week 6, NIF week 6 vs. NIF week 18) using the DESeq2 default Wald’s test. DEG’s were identified by Benjamini-Hochberg (BH)^37^ adjusted p-value < 0.05 and log2 fold change (log2FC) ≥ 1. The data was transformed using variance stabilizing transformations (Suppl. Figure 2) and non-coding genes were excluded before downstream visualization with heatmaps and gene set enrichment analysis. Gene Ontology Enrichment Analysis was performed using the clusterProfiler package^38^ and enrichGO for biological processes on two sets of DEG’s per comparison, denoted as either upregulated or downregulated based on a positive or negative log2FC. GO’s containing ≥ 3 DEG’s and BH adjusted p-value < 0.05 were determined to be significant and enriched. For Gene Set Enrichment Analysis of NIF DEG’s was performed with clusterProfiler package^37^ using the gseGO for biological process reference with p-value < 0.05 and BH adjusted p-value < 0.05 as cutoff for significant and enriched pathways. Molecular pathway dysregulation in the liver tissues was determined by Gene Set Enrichment Analysis (GSEA) surveying molecular pathways gene sets in Molecular Signature Database (MSigDB) (www.broadinstitute.org/msigdb) (Suppl. Table 4). Cross-species comparison was performed in the space of molecular pathway gene sets from Hallmark and KEGG^39^ databases (https://www.gsea-msigdb.org/ gsea/msigdb/genesets.jsp) as specified in Suppl. Figure 4 and Suppl. Tables 4, and similarity to the human datasets was determined by Euclidean distance and visualized as previously described by Tsuchida et al.^18^.

The significance values (p-value of overlap) for the IPA canonical pathways were calculated by the right-tailed Fisher’s Exact Test, and the p-values were adjusted for multiple testing using BH^37^. It was also required that at least two DEGs were associated with an enriched pathway. A ratio was calculated of the number of DEG molecules associated with a given pathway divided by the total number of molecules in the reference set that map to the pathway.

IPA also calculated for each pathway a z-score that predicted pathway activation if positive or inhibition if negative. The z-score was calculated by comparing the dataset fold changes under analysis with the canonical pathway patterns in the IPA Knowledge Base. Z-scores of ≥ _2 or ≤ _-2 are considered significant, and no z-score annotation indicates either zero (or very close to zero) z-score or that the given pathway is ineligible for a prediction.

The IPA Molecule Activity Prediction (MAP) tool was used for making predictions of the activation or inhibition statuses of interacting molecules. Diagrams were extended by adding connections to the ten most significantly associated diseases and functions or the ten most significantly associated liver-related functions.

## Electronic supplementary material

Below is the link to the electronic supplementary material.

Supplementary Material 1

Supplementary Material 2

Supplementary Material 3

Supplementary Material 4

Supplementary Material 5

Supplementary Material 6

## Data Availability

The data that support the findings within this paper and other findings of this study are available from the corresponding author upon reasonable request. The datasets generated and analyzed during the current study are available in the Gene Expression Omnibus (GEO) (GSE25526).
