# Supplementary material for: Structure-function analysis of time-resolved immunological phases in metabolic dysfunction-associated fatty liver disease (MASH) comparing the NIF mouse model to human MASH
Source: Sci Rep. 2024 Oct 3;14:23014. doi: 10.1038/s41598-024-73150-z (PMC11452201; doi:10.1038/s41598-024-73150-z)
Supplement: Supplementary file 5 — Supplementary Material 5 [file 41598_2024_73150_MOESM5_ESM.docx]

**Supplementary materials**

**Structure-function analysis of time-resolved immunological phases in metabolic dysfunction-associated fatty liver disease.**

Anja Schmidt-Christensen^1^, Gustaw Eriksson^2^, William Michael Laprade^3^ and Behnaz Pirzamanbein^3,4^, Maria Hörnberg^5^, Kajsa Linde^5^, Julia Nilsson^1,5^, Mark Skarsfeldt^6^, Diana Julie Leeming^6^, Rajmund Mokso^7^, Mariana Verezhak^8^, Anders Dahl^3^, Vedrana Dahl^3^, Kristina Önnerhag^9^, Massoud Rezaee Oghazi^10^, Sofia Mayans^5^ and Dan Holmberg^1,5,11^

^1^Lund University Diabetes Center, Lund University, Lund Sweden; ^2^Karolinska Institute, Stockholm, Sweden; ^3^Technical University of Denmark, DTU; ^4^Statistics department, Lund University, Lund Sweden; ^5^Inficure Bio AB, Umeå Sweden; *^6^*Nordic Bioscience A/S, Herlev, Denmark; ^7^MAXIV laboratory, ^8^Paul Scherrer Institut, Villigen, Switzerland; ^9^Skåne hospital, Malmö, Sweden; ^10^Connected Pathology, Belgium; ^11^Department of Medical Biosciences, Umeå University, Umeå Sweden

**Contents:**

Page

Suppl. Fig. 1 02

Suppl. Fig. 2 04

Suppl. Fig. 3 05

Suppl. Fig. 4 06

Suppl. Fig 5 08

Suppl. Table 1a DEGs control mice 3 vs. 6 w Excel file

Suppl. Table 1b DEGs control mice 6 vs. 18 w Excel file

Suppl. Table 1c DEGs control mice 3 vs. 18 w Excel file

Suppl. Table 2a DEGs NIF mice 3 vs. 6 w Excel file

Suppl. Table 2b DEGs NIF mice 6 vs. 18 w Excel file

Suppl. Table 2c DEGs NIF mice 3 vs. 18 w Excel file

Suppl. Table 3a DEGs NIF mice 3 vs. 6 w age corrected Excel file

Suppl. Table 3b DEGs NIF mice 6 vs. 18 w age corrected Excel file

Suppl. Table 3c DEGs NIF mice 3 vs. 18 w age corrected Excel file

Suppl. Table 4a GEO analysis of genes found in Table S3a. Excel file

Suppl. Table 4b GEO analysis of genes found in Table S3a. Excel file

Suppl. Table 4c GEO analysis of genes found in Table S3a. Excel file

Suppl. Table 5a GSEA result NIF mice 3 vs. 6 weeks Excel file

Suppl. Table 5b GSEA result NIF mice 3 vs. 18 weeks Excel file

Suppl. Table 5c GSEA result NIF mice 6 vs. 18 weeks Excel file

Suppl. Table 6 Animal models used for comparative analysis 09

**Suppl. Fig. 1**

**
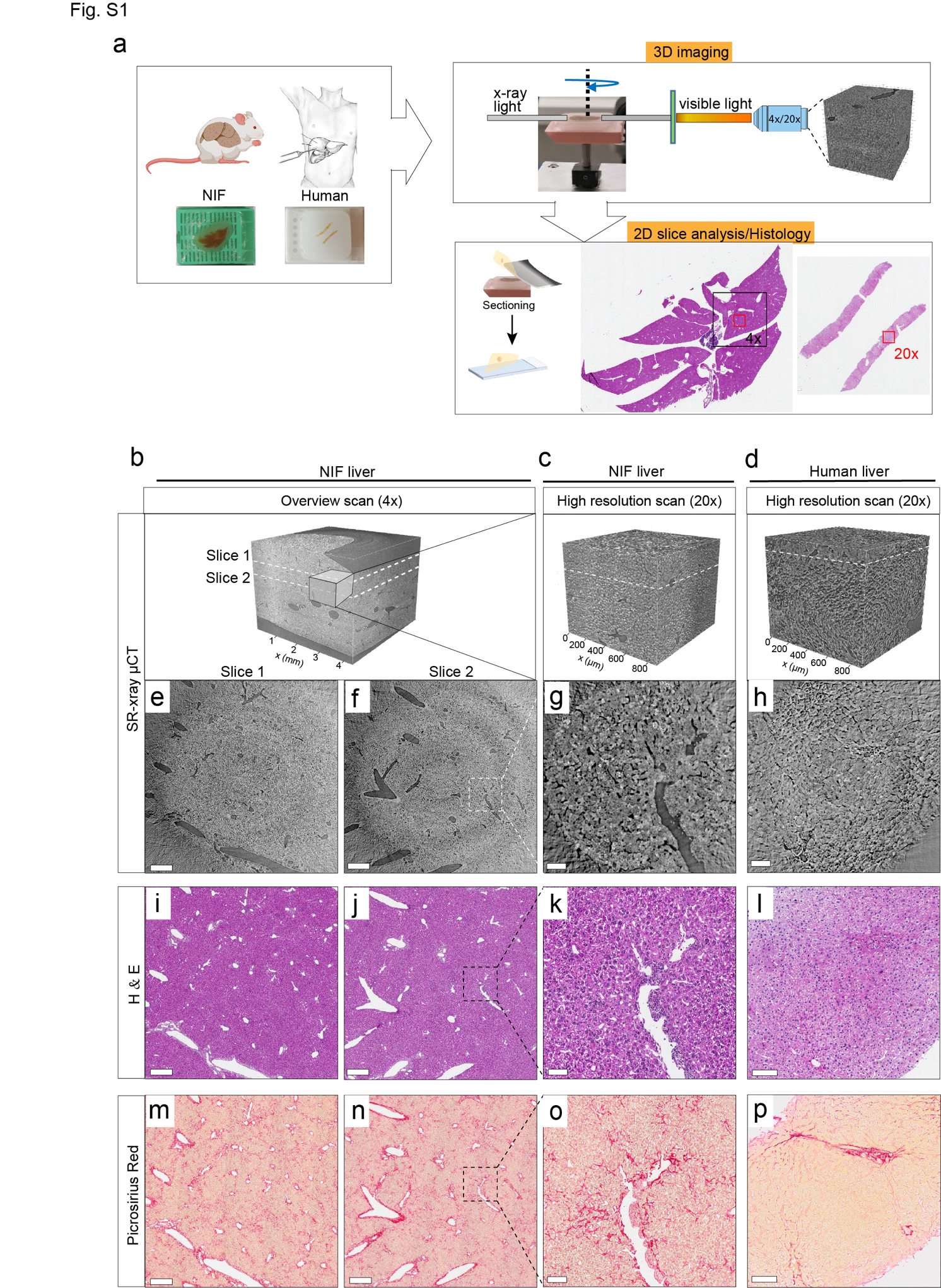
**

**Suppl. Fig. 1**: SR-µCT enables virtual histology for human biopsies and larger animal tissues. (a) Experimental setup for the structural tomographic SR-x-ray µCT imaging of fixed human liver biopsies and mouse liver samples followed by sectioning of the liver tissue blocks and conventional 2D histology for validation. Field of view for overview or high-resolution SR-x-ray µCT is indicated by black or red square for 4x or 20x magnification respectively. (b-h) Liver samples obtained from 12-week old NIF mice (b,c, e-g) or human healthy volunteers (d,h) were subjected to overview (b,e,f) and/or high-resolution SR-µCT imaging (c,d,g,h). 3D-renderings (b-d) or 2D cross-sections (e-h), as indicated in b-d, matched with corresponding histological sections stained with Hematoxylen and Eosin (i-l) or Picrosirius Red (m-p). Scale bar: 500 µm (e,f,i,j,m,n) or 100µm (g,h,k,l,o,p).

**Suppl. Fig. 2**


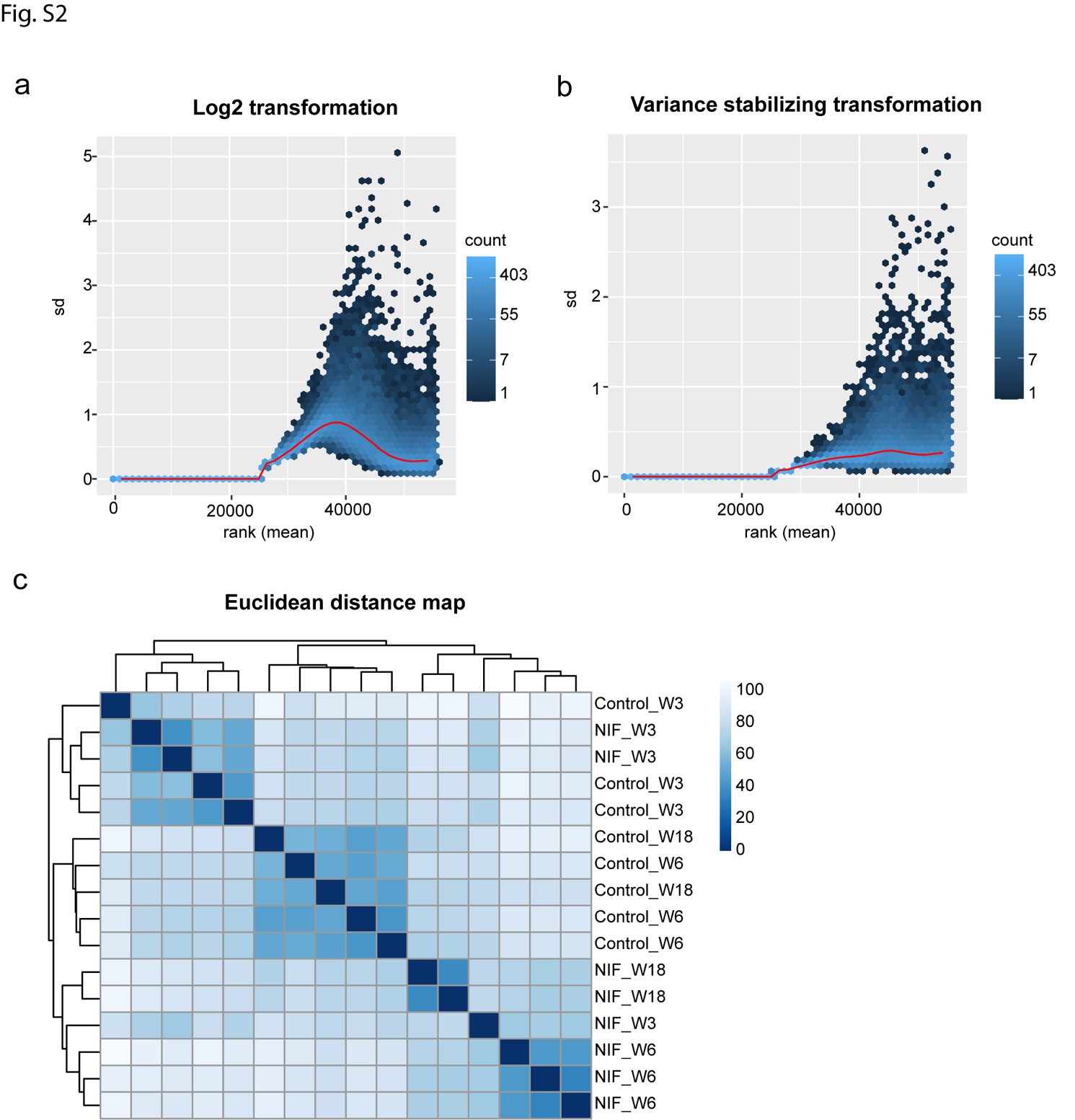


**Suppl. Fig. 2:** The standard deviation (sd) and ranked mean are calculated from the gene expression matrix row-wise per gene across all column-wise samples, to generate the scatterplot (a-b) after log2 normalization (a) and variance stabilizing transformation (b). The red line in (a-b) indicates the running median estimator of the gene expression matrix and the scale bar and dot color indicate the mean count. Euclidean distances between gene expression matrices with hierarchical clustering are presented in (c) to present similarity between the samples shown in the scale bar where 0 is similar and 100 is dissimilar Euclidean distance.

**Suppl. Fig. 3**


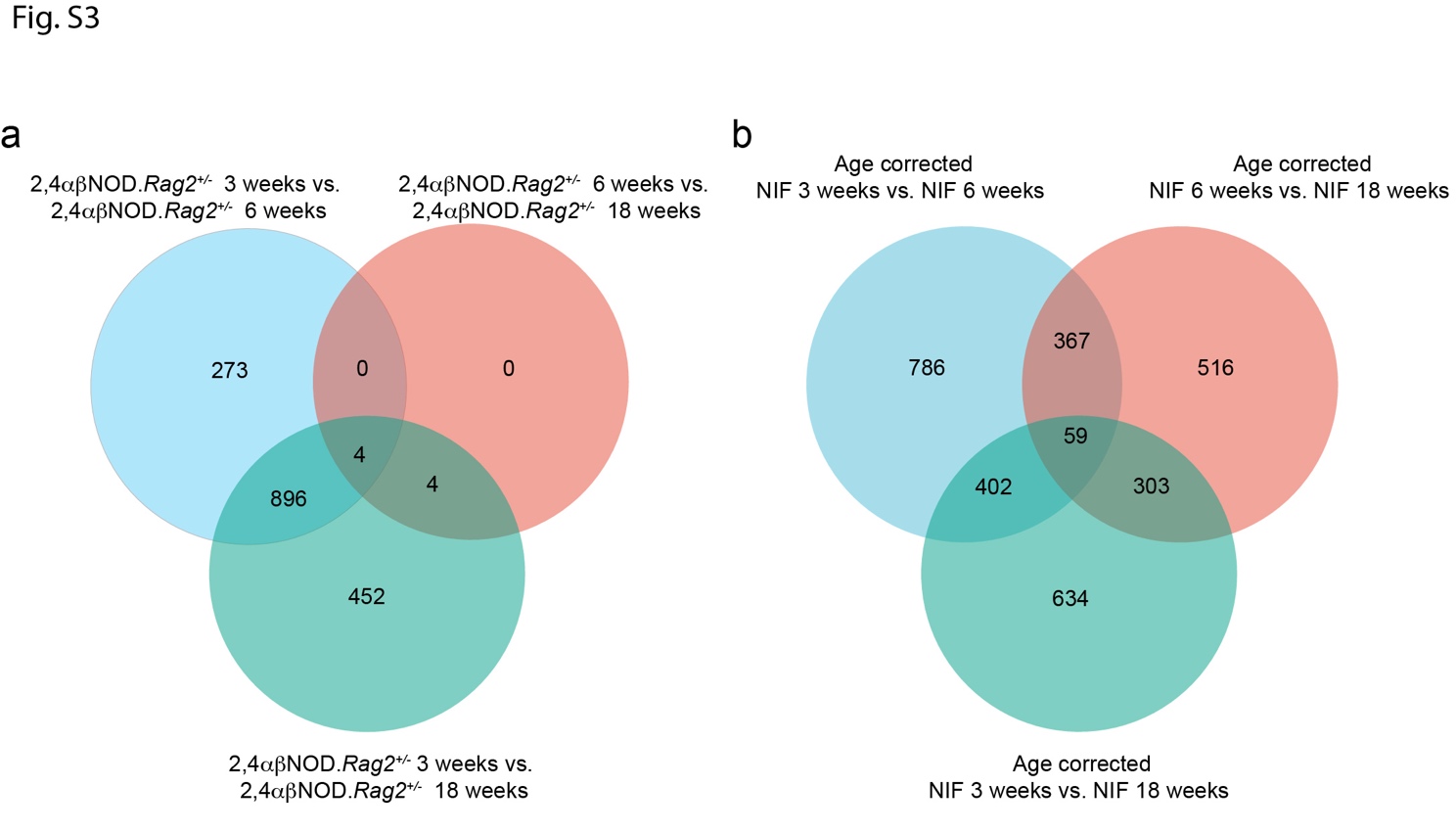


**Suppl. Fig. 3**: Venn diagrams indicating shared and unique differentially expressed genes (DEG) in liver transcriptomes across ages of 24αβNOD.*Rag2^+/-^* control (a) and NIF mice corrected for age related DEGs (b), that have been identified in (a). Timepoints were strategically selected to capture disease progression (related to Fig.4), including pre-disease onset at 3 weeks, early-stage phenotype at 6 weeks and late-stage.

**Suppl. Fig. 4.**


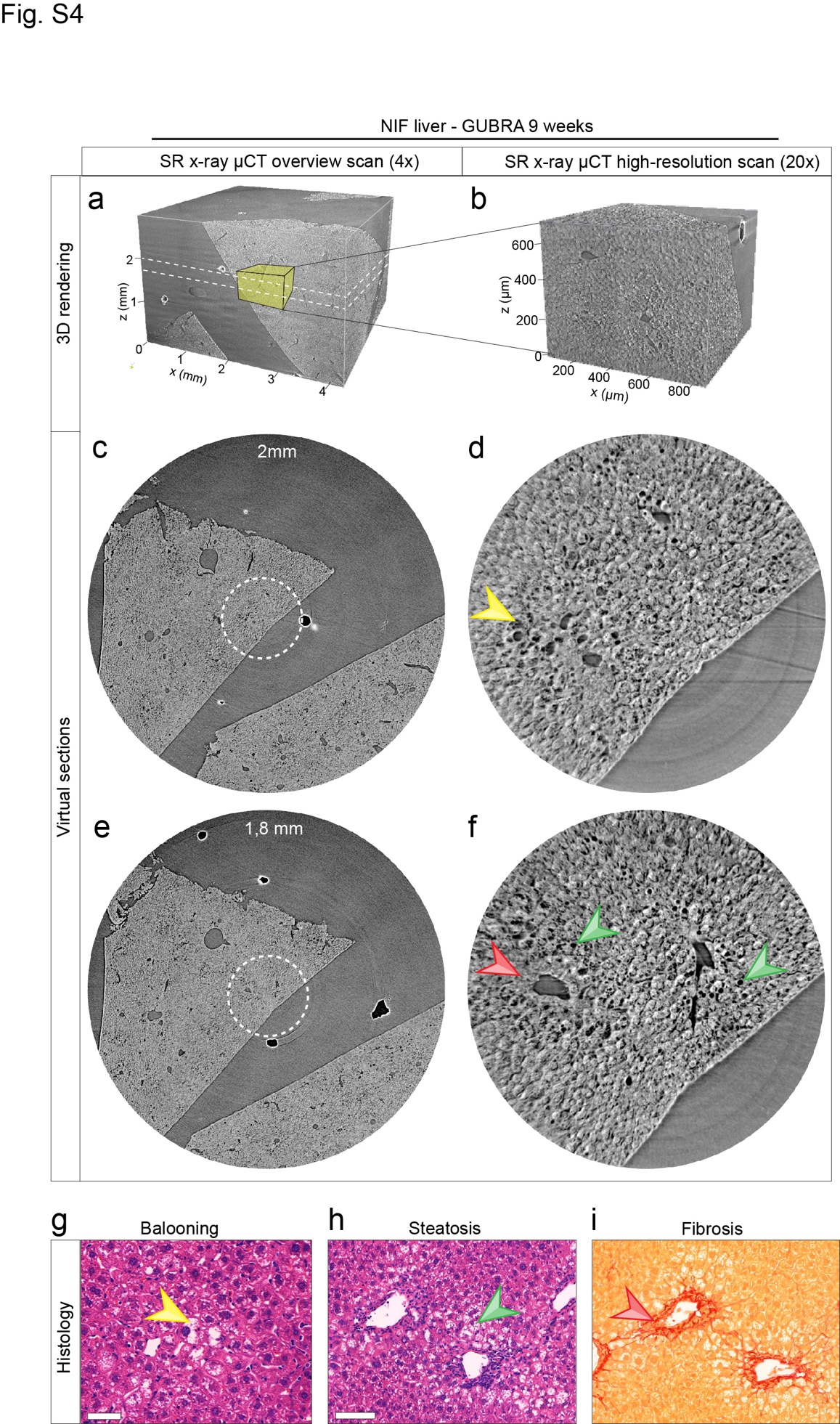


**Suppl. Fig. 4**: SR-μCT imaging reveals characteristics of NASH in GAN-diet fed NIF mice. Liver samples obtained from 12-week-old male NIF mice that have been fed GAN diet for 9 weeks were subjected to both overview (a, c, e) and high-resolution SR-µCT imaging (b, d, f) (n=3 mice). 3D-renderings (a, b) or 2D cross-sections (c-f) and Field-of-view for the corresponding high-resolution scan as indicated in (a, c, e). (g-i) Corresponding histological sections stained with Hematoxylin and Eosin (g, h) or Picrosirius Red (i). Notable structures and features are labelled: fat cell denoted by green arrow (f, h), ballooning hepatocyte by yellow arrow (d, g) and fibrosis by red arrow (d, i). Scale bar 100µm (g-i).

**Suppl. Fig. 5**

**
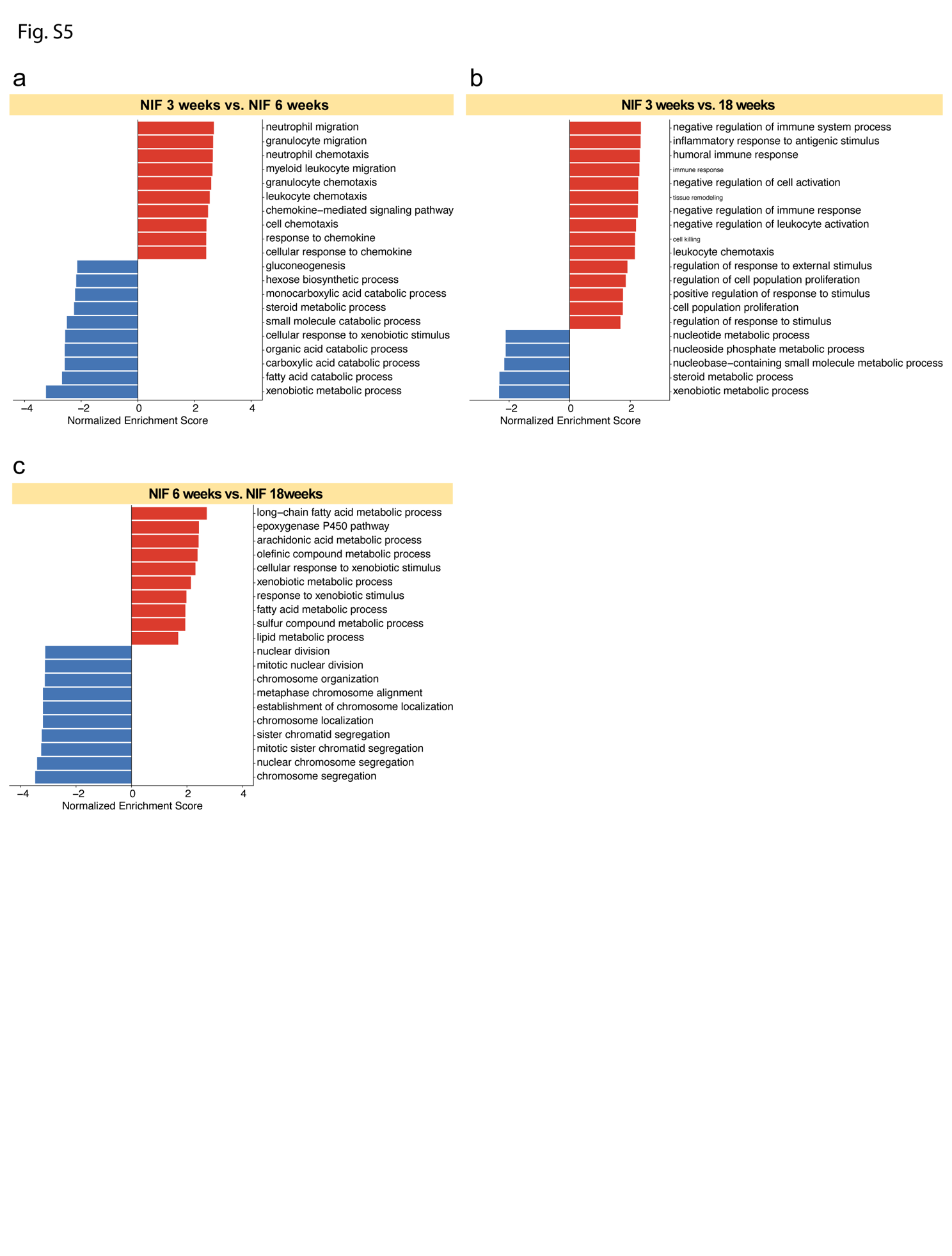
**

**Suppl. Fig. 5:** GSEA assessing gene expression in liver tissues of NIF mice at 3, 6, and 18 weeks (n=3 mice, n=3 mice, n=2 mice, respectively). Top 10 enriched pathways and gene counts for 3 vs. 6 weeks (**a**), 3 vs. 18 weeks (b), and 6 vs. 18 weeks (c) are displayed. Rank gene sets are based on significance (adjusted p-value <0.05) after age-effect corrections. Orange and green indicate upregulated and downregulated gene expression.

**Suppl. Table 6 GSEA comparison mouse models**

| **GSE Acession number** | **Tissue** | **Model** |
| --- | --- | --- |
| 48452 | Liver (human) | NASH / CTRL |
| 49541 | Liver (human) | Advanced / Mild NAFLD |
| 22608 | Liver (mouse) | OB / WT |
| 27713 | Liver (mouse) | KO / WT |
| 35961 | Liver (mouse) | NASH Metformin / Normal |
| 38013 | Liver (mouse) | SHPKO West Diet / Chow |
| 38141 | Liver (mouse) | West Diet / Control Diet |
| 39594 | Liver (mouse) | High fat / Normal |
| 59042 | Liver (mouse) | High fat / Normal |
| 62362(a) | Liver (mouse) | WSB-CFD 12Week / CTRL |
| 62362 (b) | Liver (mouse) | AJ-CFG 12Week / CTRL |
| 62362 (c) | Liver (mouse) | C3H-CFD 12Week / CTRL |
| 63027 (a) | Liver (mouse) | *gnmt* KO / WT |
| 63027 (b) | Liver (mouse) | *mat1a* KO / WT |
| 67680 | Liver (mouse) | West Diet / Control Diet |
| 70681 | Liver (mouse) | Mutant / WT |
| 83596 | Liver (mouse) | Tumour / Non-Tumour |
| 99010 (a) | Liver (mouse) | WDCCL4 12Week / CTRL |
| 99010 (b) | Liver (mouse) | WDOil 12Week / CTRL |
| 99010 (c) | Liver (mouse) | WDOil 24Week / CTRL |
| 99010 (d) | Liver (mouse) | WDCCL4 24Week / CTRL |
| 99010 (e) | Liver (mouse) | NDCCL4 12Week / CTRL |
| 99010 (f) | Liver (mouse) | NDCCL4 24Week / CTRL |
