# Supplementary material for: Structure-function analysis of time-resolved immunological phases in metabolic dysfunction-associated fatty liver disease (MASH) comparing the NIF mouse model to human MASH
Source: Sci Rep. 2024 Oct 3;14:23014. doi: 10.1038/s41598-024-73150-z (PMC11452201; doi:10.1038/s41598-024-73150-z)
Supplement: Supplementary file 6 — Supplementary Material 6 [file 41598_2024_73150_MOESM6_ESM.docx]

Legends to supplementary videos

**Suppl. Movie 1**: 3D-rendering of a high-resolution SR-µCT liver scan (20x) from an 8-week-old NIF mouse, RAW-data. Similar cellular resolution in x,y,z allow virtual dissection and flexible viewing from any angles. Vessels and sinusoids (paraffin-filled) appear dark. Inflammatory cells within lesions are visible as lighter grey dots. Related to Fig. 2.

**Suppl. Movie 2**: 3D-rendering showing segmented features from a high-resolution SR-µCT liver scan (20x) from an 8-week-old NIF mouse (related to Suppl. Video 1 and Fig. 2). Lesion regions are visualized in red, disrupted sinusoidal structures within lesion regions appear in beige and sinusoidal structures within unaffected healthy regions appear green.

**Suppl. Movie 3**: 3D-rendering showing segmented features from a high-resolution SR-µCT liver scan (20x) from an 8-week-old 24αβNOD.Rag2+/− control mouse (related to Fig. 2). Sinusoids are visualized in green and larger vessels green with a yellow core. Note: large vessels possess walls with high degree of collagen-fibers and can be detected by the segmentation tool as minor “lesion” (red) around vessels.
